# A rare case of intracranial solitary fibrous tumor that is still alive after multiple surgical resections: a case report and review of the literature

**DOI:** 10.3389/fneur.2023.1201964

**Published:** 2023-07-10

**Authors:** YiMeng Gao, Yan Xu, RuiZhi Xie, YouBi Shen, DaoJin Xue, Zheng Zhen, JingJing Lu, Tao Huang, ZiZhuang Peng

**Affiliations:** ^1^The Second School of Clinical Medicine of Guangzhou University of Chinese Medicine, Guangzhou, Guangdong, China; ^2^Neurosurgery, Guangdong Provincial Hospital of Traditional Chinese Medicine, Guangzhou, Guangdong, China; ^3^Acupuncture and Rehabilitation, The First Affiliated Hospital of Guangzhou Medical University, Guangzhou, Guangdong, China

**Keywords:** intracranial solitary fibrous tumor, hemangioepithelial cell tumors, gross total resection, multimodal treatment, follow-up

## Abstract

A Solitary Fibrous Tumor (SFT) is a rare, aggressive, and metastasis- and recurrence- prone mesenchymal tumor. In this case report and review, we describe a rare instance of intracranial SFT, discovered for the first time. It was discovered in 2008 and following total surgical removal, the pathology was categorized as hemangiopericytoma cell tumor (HPC) at the time by WHO tumor criteria. An imaging review 8 months after surgery revealed a tumor recurrence: combined radiation and gamma-knife therapy was continued throughout this time. The tumor did not metastasis until June 2018 when it presented in the pancreas with ruptured bleeding and a postoperative pathology was suggestive of SFT. Fortunately, the patient is still alive nearly 3 years after the 2020 surgery, after staged surgical resection and combined multimedia therapy, with no imaging or clinical evidence of a recurrent intracranial primary lesions. To our knowledge, there is no previous record of using a combined treatment modality for Intracranial Solitary Fibrous Tumor (ISFT). Combined with an account of the patient's experience, we empirically describe a combined approach with a preference for gross-total resection (GTR), supplemented by multimodal assistance with stereotactic (radiotherapy), gamma knife (GK), molecular targeting, and immunization for patients admitted acutely, with accurate preoperative identification and aggressive management after intraoperative case response to maximize treatment of recurrent ISFT and improve prognosis. We recommend multimodal management for SFT with prolonged-term recurrence and metastases, both for the control benefits of GTR, RT, or GK for local recurrence and for the positive prognosis of targeted and immune metastases.

## 1. Introduction

A Solitary Fibrous Tumor (SFT) is a mesenchymal tumor characterized by specialized fibroblasts of intermediate biological potential. It is usually found in pleura (1). Cases of SFT occurring in the central nervous system (CNS SFTs) were first reported by Carneiro and Scheithauer in 1996 (2). Another term, “hemangiopericytoma,” is used to describe an SFT and hemangiopericytoma cell tumor (HPC), which has been categorized as a distinct entity according to World Health Organization (WHO) CNS tumor classification criteria since 2016. Since an increasing number of studies have demonstrated that SFTs and HPCs have similar immunohistochemical profiles, they are now collectively referred to as SFTs by the WHO (2021). Among all cases reported to date, intracranial solitary fibrous tumors (ISFTs) are rare, accounting for ~1%−4% of intracranial tumors (3). Female and male patients are equally likely to be affected, with onset generally occurring between the age of 51 and 60 (4). SFTs are more likely to recur and metastasize compared with other tumors. Based on follow-up data, the rate of recurrence is as high as 75% after 10 years of disease (5). The rate of extracranial metastasis is ~13–55% (the metastasis rate was about 11.1–57%) (6), and the median survival rate after metastasis is between 22 and 46 months (7).

Currently, the main clinical treatment is based on GTR that prioritizes neurological functions, supplemented by multi-mode therapy such as stereotaxis (radiotherapy), GK, molecular targeting, and immunization, etc. In this article, we describe a rare and exceptional case of ISFT active for up to 14 years, with multiple, recurrent, and systemic metastases is presented, including the patient's complete medical history and clinical trajectory. By collecting and studying relevant literature, further information on nomenclature grading, recurrence and survival, diagnosis, multimodal treatment, and prognosis of ISFT is discussed. The purpose of this article is to provide some references and suggestions for the management of these rare clinical cases.

## 2. Case

**Figure 1:**
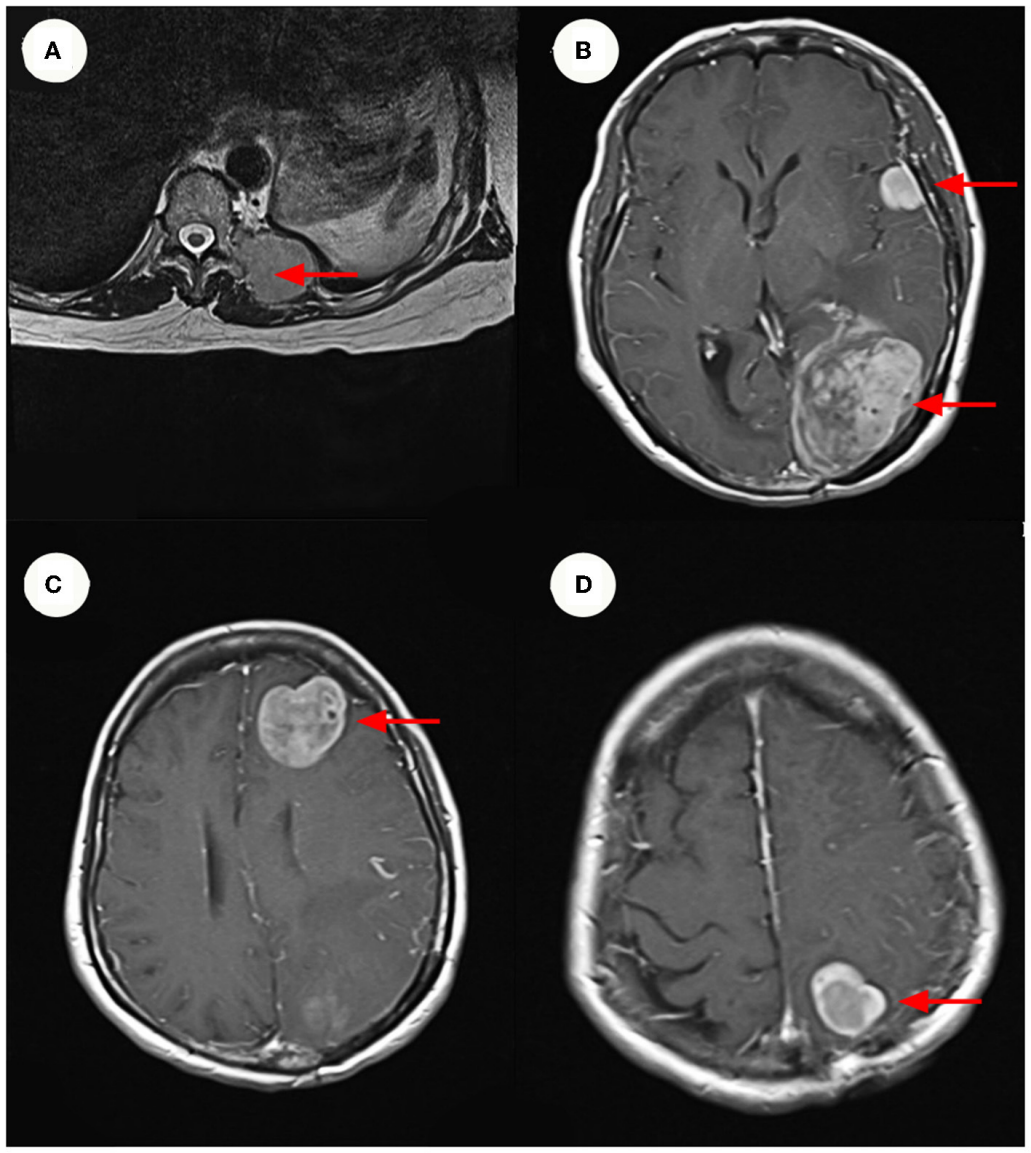
MRI and Enhanced MRI images. **(A)** Show hyper-intense signal in the patient's lumbar MRI images. The size of the mass ~3.3 x 4.3 cm. **(B–D)** Show hyper-intense signal in the patient's cranium Contrast-Enhanced MR imaging. The largest signal shadow was found in the left occipital lobe, measuring ~5.3 x 4.1 cm. It was accompanied by a ruptured hemorrhage, some of which entered the left lateral ventricle. **(B–D)** Are axial images, with the red arrow indicating the mass.

A 64-year-old female patient visited the orthopedic department of Guangdong Provincial Hospital of Traditional Chinese Medicine in China on October 7, 2020, due to repeated left lower extremity paresthesia and limited movement. The patient was in a normal state of consciousness during the examination, while declining cognitive, speech function, and the muscle strength of the right limb was 5-grade. Meanwhile, the magnetic resonance imaging (MRI) of the lumbar spine (Figure 1) showed a soft tissue mass shadow on the left side of the T11 adnexa. A cranial MRI (Figure 1) also revealed several abnormal signal shadows in the left frontal lobe and bilateral parieto-occipital lobes. The patient underwent a complete positron emission tomography (PET) scan that revealed multiple metastases throughout the body (Table 1).

**Table 1:** The location and times of metastases in this case of malignant SFT.

**Time**	**Diagnosis**	**Location**	**Size**
Oct 2020	Systemic widespread secondary malignancy (SFT)	Both lungs	1.3 cm (Multiple)
		Left posterior 10th rib	5.2 x 5.1 cm (Maximum cross section)
		L2 vertebra	/
		Right sacrum	/
		Bilateral iliacus	/
Mar 2021	Systemic widespread secondary malignancy (SFT)	Subsurface S6 envelope of the liver	2.9 x 2.5 cm
		Right lower abdominal wall	/
		Left superior femur	Multiple

SFT, solitary fibrous tumor; WHO, World Health Organization.

In 2008, the patient had received the first treatment that was an excision of the left occipital region 14 years previously, which had been identified as having an HPC of grade II-III. Radiation therapy (RT) was then chosen by the patient after surgery. After 2 months, no sign of recurrence was discovered in the follow-up examination. Nevertheless, in March 2009, a recurrence was detected in the primary focus. The patient underwent GK following advice from an external neurosurgeon. In follow-up examinations, neither the patient's clinical symptoms nor the tumor size increased significantly. In February 2018, the patient received GK (left frontal and left temporal lesions, 40% isometric curve, 14–16 Gy peripheral dose, 35–40 Gy central dose).

**Figure 2:**
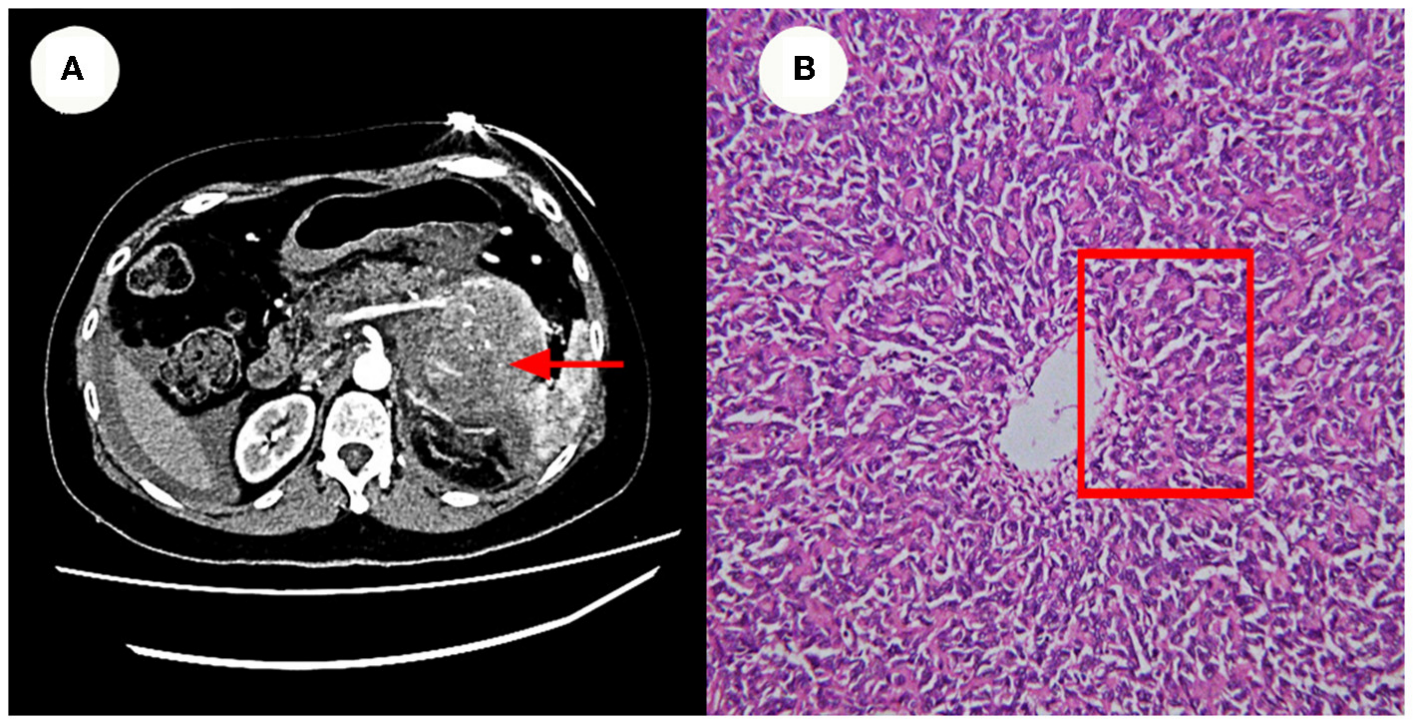
Enhanced CT and Pathology images. **(A)** Show a caudal pancreatic occlusion combined with bleeding (~6.1 x 13.5 x 8.0 cm in size). **(B)** Shows that the cells were also short spindle or ovoid in shape, with inconspicuous nucleoli and 3–5 nuclear fission images/10 HPF. Its features at red rectangle.

A partial pancreatectomy and splenectomy were performed on the patient in our hospital in June 2018 for a caudal pancreatic occlusion combined with bleeding (Figure 2). The immunohistochemical results (Figure 2) were positive for Vim, SMA, and B-catenin (plasma), had a focal positive result for CD34, CD99, and S-100, a weak positive result for ERG, Bc1-2, and FLI-1, and a negative result for CD117, CD56, STAT6, and Ki67 index 10%. The pathological findings at that time suggested atypical and malignant SFT (metastases).

**Figure 3:**
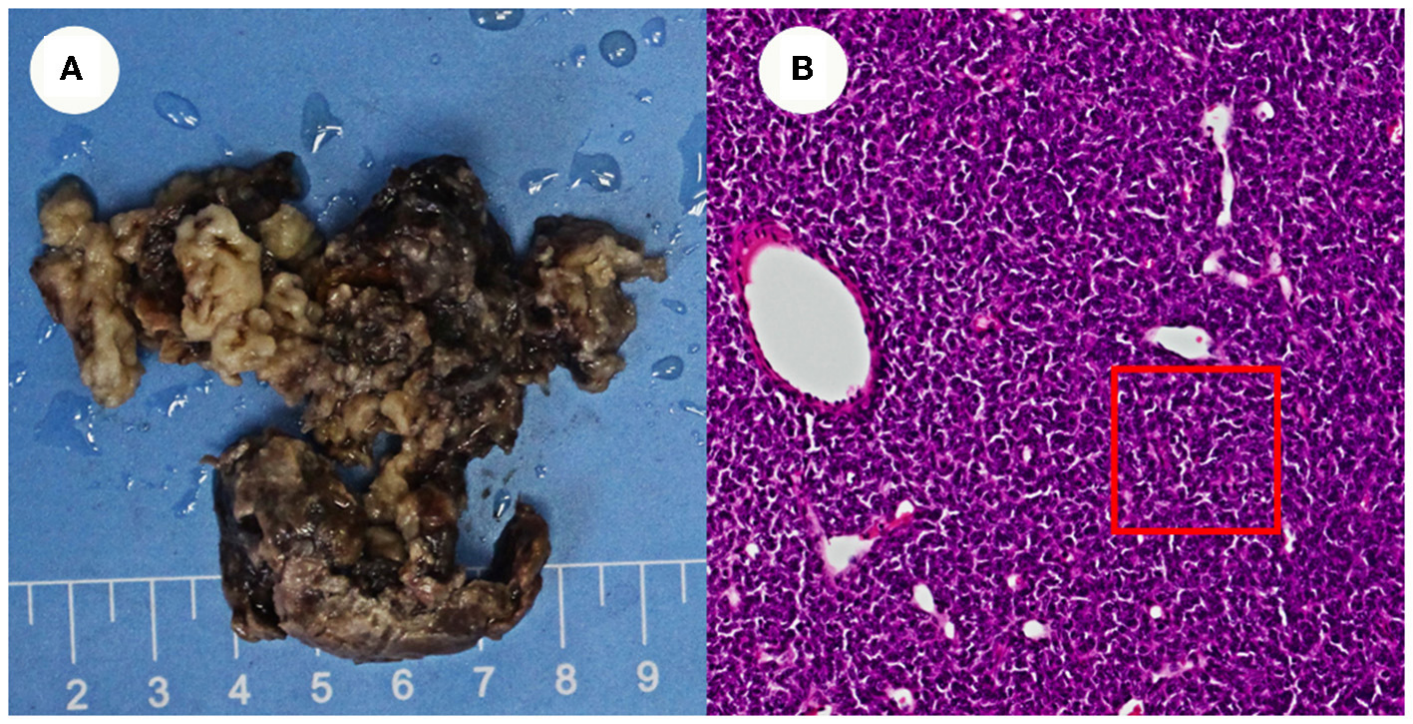
Appearance of the resected tissue and pathology images. **(A)** Shows the swelling was fish-like in appearance, gray in color, and slightly soft in texture. **(B)** Shows cranial pathology imagines.

In 2020, a mass in the lumbar spine was investigated as a possible metastatic tumor based on the patient's medical history. The patient was transferred from orthopedics to neurosurgery because of the complex conditions, and three frontotemporal tumors were removed. The swelling was fish-like in appearance, gray in color, and slightly soft in texture (Figure 3).

**Figure 4:**
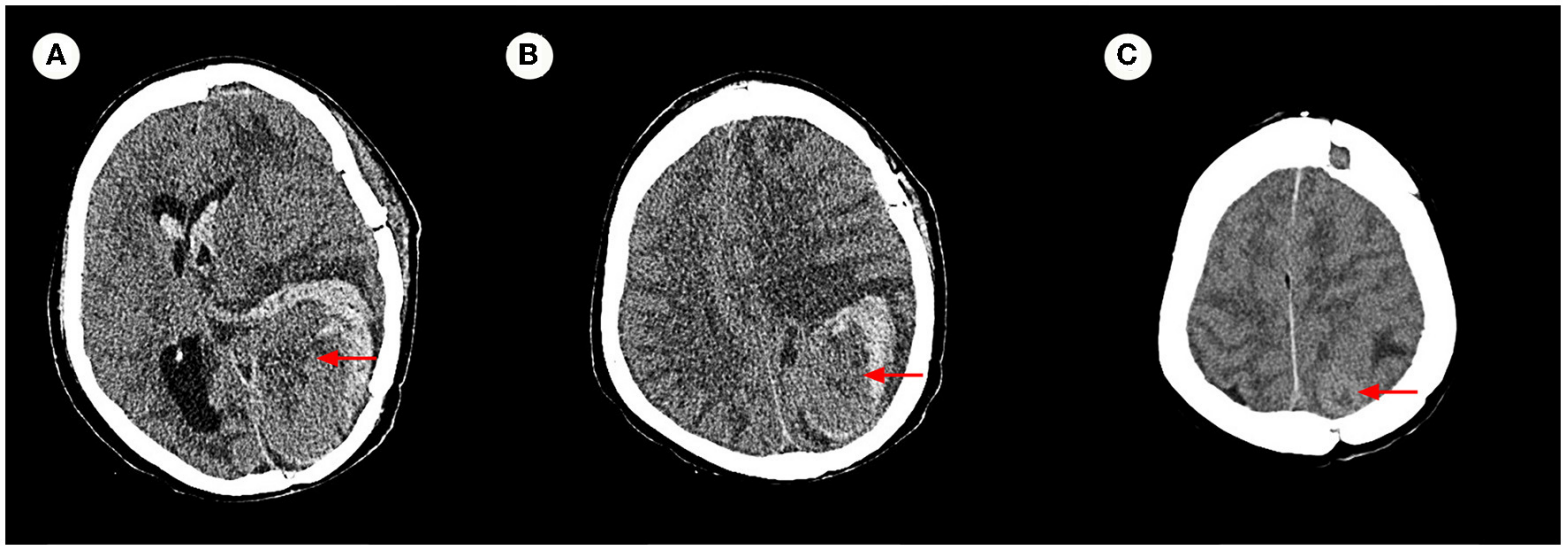
Axial cranial CT image. **(A–C)** Confirmed that the left remaining occipital lobe mass (measuring 6.7 × 4.2 cm), with increased surrounding hemorrhage, and herniation formation of the left temporal lobe hook gyrus and parahippocampal gyrus.

**Figure 5:**
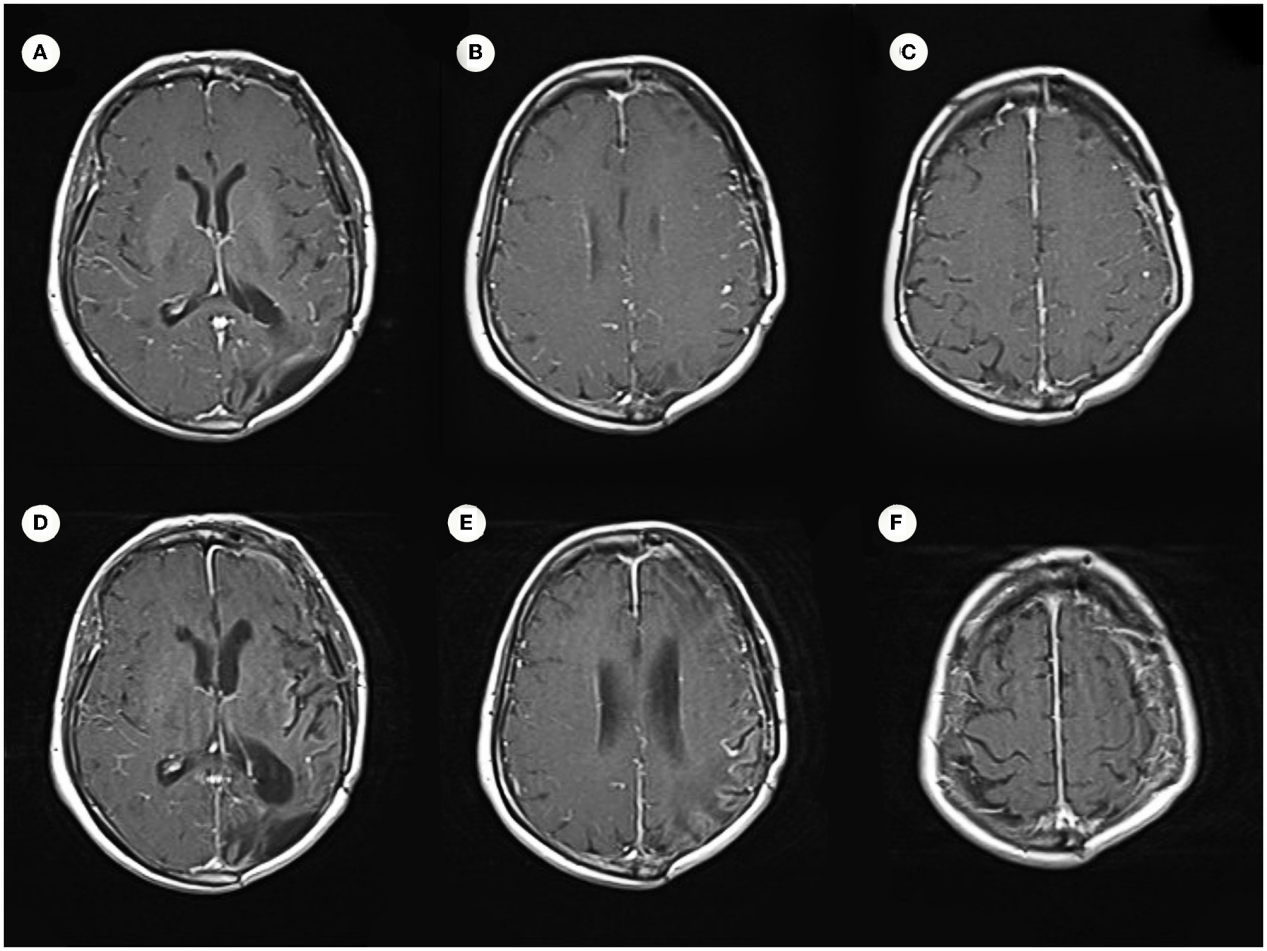
Enhanced MRI images. **(A–C)** Are consistent with postoperative total resection. **(D–F)** Show no obvious signs of recurrence of intracranial lesions in a recent review.

The pathology of the tumor cells revealed that cells were solid and grew in a lamellar way (Figure 3). The cells were also short spindle or ovoid in shape, with inconspicuous nucleoli and 6–15 nuclear fission images/10 HPF. Immunohistochemical staining suggested positive results for Vim, ERG (vascular), and CD99, and had scattered or localized positivity for S-100, CD34, NSE, and STAT6. The Ki67 index increased significantly to 15%. According to the 2016 WHO classification system for tumors of the central nervous system and in conjunction with the pathology, the final diagnosis was ISFT (WHO grade III). The patient was hospitalized on November 11, 2020, after experiencing a sudden onset of confusion. Emergency cranial computed tomography (CT) confirmed the left remaining occipital lobe mass (Figure 4). We performed total resection of the tumor. The pathological nature of the tumor was like that of the previously resected tumor, which was consistent with SFT, and postoperative MRI confirmed total tumor resection (Figure 5). Neurologically, the patient improved significantly following the operation, and muscle strength and tone returned to normal. The patient was discharged from the hospital for regular follow-up CT/MRI imaging. Afterward, liver metastasis and metastasis in the right lower abdominal wall were found by abdominal CT in March 2021 (Table 1). Considering the patient's complicated medical history, including multiple metastases throughout the body, several surgeries, and recurrence, as well as the fact that the patient had received surgery and radiation, Anlotinib targeted therapy (March 2021) and Sintilimab immunotherapy (May 2021) were given by the oncologists consecutively. The patient's treatment was well-tolerated, with no particular adverse reactions, and they are currently stable and progressing slowly. During follow-up in July 2021, the patient was treated with GK again in another hospital, and the general condition remained stable, with no obvious signs of recurrence of intracranial lesions (Figure 5).

Earlier medical records cannot be retrieved due to the complexity of the patient's medical history, the long-time span, and unsolvable storage issues related to medical equipment. Applying the data in Table 2, an attempt was made to review the patient's entire course of illness.

**Table 2:** Timeline of the patient's illness.

**Time**	**Tumor location**	**Size**	**Treatment**	**Immuunohistochemistry (+)**	**WHO grade**
Jul 2008	left occipital	6.9 x 4.4 cm	GTR+RT	CD34,Vim	II-III
Mar 2009	left occipital (Recurrence)	1.5 x 2.0 x 1.9 cm	GK	N,D	N,D
Feb 2018	left frontal, left temporal lesions	N,D	GK	N,D	N,D
Jun 2018	Pancreas	6.1 x 13.5 x 8.0 cm	GTR	CD34, B-catenin, Vim, SMA, CD99, S-100	III
Dec 2018	left frontal lobe	0.4 x 0.7 cm	N,D	N,D	N,D
	Left temporal lobe	0.4 x 0.5 cm	N,D	N,D	N,D
Aug 2019	Superior sagittal sinus	1.4 x 2.7 x 1.3 cm	RT	N,D	N,D

GTR, gross total removal; RT, Radiotherapy; N,D, no data; WHO, World Health Organization; CD34, cluster of differentiation 34; CD99, cluster of differentiation 99; Vim, vimentin; S-100, s-100 protein; SMA, Smooth muscle actin.

## 3. Discussion

### 3.1. Naming and grading

There is a long history of coexistence between SFT and HPC in literature. Lietaud first mentioned SFT in the pleura in 1767 (8), and Stout and Murray proposed HPC in 1942 (9). According to the 2007 WHO classification of central nervous system tumors, HPC and SFT are distinct lesions (10). By 2012 the WHO classification of soft tissue and bone tumors integrated SFT and HPC into one disease entity (11). New research in 2013 revealed that SFT exhibits features of HPC histology and immunohistochemistry. Moreover, NAB2-STAT6[(nerve growth factor induced gene A binding protein 2)—signal transducers and activators of tranion 6, STAT6)] gene fusions with translocations on chromosome 12q13 have frequently been detected (12). It was not until 2016 that low-grade SFTs, high-grade HPCs, and interstitial HPCs were considered to be a single category, SFT/HPC, in the WHO classification (13). The terminology “SFT” was applied in WHO classification in the latest 2021 edition, with “HPC” removed (14). According to statistics, SFT accounts for ~2% of all primary soft tissue tumors (1). SFT occurring in the CNS SFT accounts for only 1% of all primary tumors, and intracranial SFT is even more rare (5).

As for the case mentioned, the patient was first diagnosed with HPC grade II–III 14 years ago (2008). After a thoughtful study of the historical complexity of the relationship between SFT and HPC, as well as their eventual unification, a reevaluation of the pathological tissue was obtained during the patient's first surgery (2008), which discovered that the characteristics of the tissue were consistent with the current WHO-SFT grade III criteria. Since no metastases were found on examination at that time, and even though the progression of systemic multiplication was discovered over the subsequent 14-year course of the disease, it is reasonable to assume that the main focus was intracranial.

### 3.2. Diagnosis

The diagnosis of ISFT was mainly based on pathology or immunohistochemistry results, with imaging and other tests serving as supplementary diagnostic measures. Pathologically, SFT sections were found to exhibit color changes primarily due to collagen fibers, blood vessels, necrosis, and mucus-like or cystic degeneration. The tumor cells mainly consisted of hyaline or sclerotic collagen fibers arranged in short, randomly oriented bundles, whorls, or nests of predominantly oval or spindle-shaped cells. Moreover, in the tan flesh in cellular tumors or gelatinous in mucinous tumors, the presence of distinct hemorrhagic domains as well as necrosis can be observed (3, 15). For immunohistochemistry, Schweizer et al. stated in 2013 that the new genotype of NAB2-STAT6 served as the most specific diagnostic marker, possessing a sensitivity and specificity of 97% (12). Yoshida et al. (16) performed STAT6 staining on 49 SFTs in 2014 and demonstrated that 100% were diffusely expressed. In the course of developing immunohistochemical techniques, additional markers that seem to be associated with SFT have been discovered. To date, the immunohistochemical markers CD34, BCL-2, wave protein, and CD99 are almost always expressed positively or strongly positively in SFT (17). However, occasionally SMA is also positive (15). Furthermore, keratin, actin, junctional protein, epithelial membrane antigen, S-100, BEGF, and EMA are often negative (3). It has been reported that CD34 is highly sensitive to SFT, with expression rates of 90–95% (18). Additionally, the positive expression rates of CD99 and bcl-2 are 75%−100% and 80%−100%, respectively (19). Multiple studies are necessary to validate their potential role. In addition, some recent studies have conducted genetic and molecular analyses of dysregulated angiogenesis for SFT. They found that upregulated of Vascular Endothelial Growth Factor (VEGF) and its receptor VEGFR were associated with activation of the AKT pathway. From this perspective, the tumorigenesis or origin of SFT may be related to the activation of angiogenic signaling pathways (20). We believe these findings provide more possibilities for the diagnosis of SFT.

### 3.3. Recurrence and survival

A high recurrence rate and high tumor mortality are associated with SFT/HPC, which is classified into three grades by the 2016 WHO classification system. Grades II and III of these are more likely to have malignant behavior, as they have greater recurrence rates, extracranial metastases, and higher fatality rates **[**3]. There have been reports of tumors metastasizing anywhere from 2 to 18 years after resection. The most common is bone metastases with a rate of 19.6%, followed by lung and pleura metastases with a rate of 18.4%, liver with 17.6%, and vertebrae with 14.1% (21). The median survival of patients with extracranial metastases is only 4.4 years after the occurrence of extracranial metastases (3, 6, 22), of which the rate has been found to be about 13~55% in some literature (3, 23–25). Moreover, a retrospective study (26) found that CD34 negativity was more likely to indicate the presence of head and neck metastases. In this case, the patient was first diagnosed intracranially with SFT 14 years ago (2008), and by 2020, metastases were in the pancreas, lung, liver, bone, and other organs. It is rare for a patient to survive multiple surgeries, radiotherapy, targeted therapy, immunotherapy, and other systemic combination therapies and still be in stable condition.

### 3.4. Points to consider

In total, 180 pertinent papers were found after screening the literature published in PubMed between 1996 and October 2022 using the common search term Intracranial Solitary Fibrous Tumor (ISFT). The types of literature collected included retrospective analyses and case reports, etc. Three cases were selected after other literature being excluded due to them having incomplete case information and <10 years of survival. The statistical results are presented in Table 3. In light of the literature and the facts of the patient, the following proposition is presented.

**Table 3:** Review of previously reported cases of ISFT with survival time≥10 years from 1996 to 2022.

**Author**	**Year**	**Age (years)/gender**	**Tumor location**	**Immuunohistochemistry(+)**	**Treatment**	**Recurrence(m)**	**Follow up (m)**	**Outcome**
Oliveira et al. (27)	2017	59/M	Lt temporal lobe	CD34, STAT6, Bcl-2, Vim	GTR+RT	34	178	S
Shirakura et al. (28)	2022	27/F	Rt posterior cranial fossa	CD34	GTR	N	288	S
		35/F	Lt posterior fossa	N, D	N,D	Multiple	264	D

M, Male; F, Female; m, month; N, No; Rt, Right; Lt, Left; N, D, no data; D, Dead; S, survival; GTR, Gross total resection; RT, RadioTherapy; CD34, Cluster of differentiation 34; Bcl-2, B-cell lymphoma 2; STAT6, Signal transducer and activator of transcription 6; Vim, vimentin.

#### 3.4.1. Multimodal treatment

Because of ISFT's low occurrence, evidence for the general treatment of ISFT relies on limited case reports and retrospective studies, and there are no definitive guidelines for the treatment of ISFTs, especially concerning recurrence and metastasis (29). The GTR that prioritizes the protection of neurological function is the primary treatment for ISFT. To obtain optimal results, the surgical plan and treatment strategy need to consider the patient's clinical symptoms and imaging characteristics. The progression-free survival (PFS) and overall survival (OS) of GTR are generally better than those of subtotal resection (STR) and partial resection (PR) (30). However, surgical resection has uncertain limiting factors. For example, to obtain the optimal surgical program suitable for the patient, most surgeons need to select the method of tumor resection based on the patient's symptoms, imaging features, tumor location, and other factors. Because this is an aggressive and malignant tumor. Surgical resection cannot prevent recurrence or metastasis in ISFT patients. Therefore, postoperative radiotherapy (PORT) is a good option for patients with ISFT. Patients often receive Intensity Modulated Conformal Radiotherapy (IMRT), stereotactic radiosurgery (SRS), and gamma knife treatment postoperatively to reduce recurrence and improve their quality of life (31–33). Stereotactic radiosurgery (especially GK) has been a promising approach for postoperative treatment of SFT over the past decade, with 3-year progression-free survival rates ranging from 60 to 92% (34–36). Multi-center studies have also reported that a cumulative radiation dose of GK is associated with the treatment of SFT (34, 35, 37). However, Due to the diversity of existing postoperative treatment modalities and the rarity of ISFT, the effect of PORT on the OS of ISFT is still being discussed. Park et al. (38) found that in patients with locally advanced, recurrent, or metastatic SFT treated with cytotoxic chemotherapy, the tumor response to chemotherapy was poor. The objective response rate was 0% and the median PFS duration, measured from the start of chemotherapy, was 4.6 months. Constantinidouet al. (39) found that more than 50% of patients progressed with non-first-line chemotherapy and a median PFS of 4.2 months in their study, which provides evidence that conventional palliative chemotherapy is of limited value. These studies have demonstrated weak sensitivity to chemotherapy for metastatic or recurrent SFT. Therefore, the efficacy of conventional chemotherapy in the treatment of recurrent or metastatic ISFT is limited. There is great potential for molecularly targeted therapies with the rapid developments in our understanding of the molecular genetics of tumors. Temozolomide in combination with bevacizumab, pazopanib, sunitinib, and radiotherapy in combination with toremifene have been reported as having been applied in the treatment of SFT (40–43). They are all beneficial to the PFS and OS of patients. Anlotinib has been reported to have potential therapeutic effects in patients with soft tissue sarcomas and various advanced cancers (44, 45). In our case, we tried to apply the targeted drug, anlotinib to control ISFT and metastases at related sites. The patient's disease is progressing slowly at present, and we believe that anlotinib may become a new choice for advanced or metastatic ISFT after future large-sample clinical studies. There are, however, some limitations to using molecularly targeted drugs, such as difficulty in screening targets and susceptibility to drug resistance. Immunotherapy is a better option for patients with intolerant advanced tumors, and future use of this approach is promising.

After being diagnosed with ISFT 14 years ago, the patient never stopped fighting the disease and actively cooperated with doctors. Aside from the surgical removal of the primary focus, the patient survived and remained in good condition by taking SRS, IMRT, targeted therapy, immunotherapy, and other treatments in the initial stage.

The patient has witnessed the evolution of SFT treatment and diagnoses over the past few decades. The clinical connotations of SFT have expanded from hypocellular fibrous SFT to stand-alone SFT combined with HPC, and then to mesenchymal SFT which tends to become sarcomatous. On reflection, timely diagnosis, regular follow-ups, and surgical procedures that could preserve neurological function as much as possible are all crucial to the treatment of the patient. Furthermore, aggressive radiotherapy, even the currently popular targeted therapies, and immunotherapy all had a significant impact on the prognosis of the patient. The case provides clinicians with more references and lessons to consider when dealing with patients with SFT.

#### 3.4.2. Prognostic factors

Kim et al. (30) concluded that the size of the tumor removed is a good predictor of the outcome of SFT. The OS and PFS were longer in the GTR group compared to the STR group according to Melone et al. (22) in their description of 43 cases (*p* = 0.047 and *p* = 0.0025, respectively). Some studies (46) found that the prognosis of a patient may depend on the condition of the interfaces of the tumor and the brain, such as the amount of cranial infiltration, the degree of attachment to the dura mater, and the grade of the tumor. According to a study on ISFT malignancy, low-grade Meningeal Solitary Fibrous Tumors (MSFTs) can transform into high-grade. The malignant progression, possibly associated with mutations in NAB2-STAT6, CDKN2/p16, or the TERT promoter gene, of high-grade is crucial to frequent relapses (47). Furthermore, by comparing programmed cell death-1 (PD-1) and its ligand-1 (PD-L1) in 16 patients with ISFT, Kamamoto et al. (48) found that PD-L1 could be diffusely or strongly expressed. Their research claims that this may lead to extracranial metastasis and that inhibiting immune check targets may be a new way to prevent metastasis, with the potential to improve the prognosis of patients. As part of this literature search, we also found reports indicating that some cases with histological features consistent with the primary lesion had slow disease progression, whereas cases with rapid progression and progression were found to have dedifferentiated histological features and loss of CD34 and Bcl-2 expression (49), with reduced or absent CD34 expression indicative of ISFT malignancy or tumor progression (50). The differential expression of these molecular gene proteins may indicate a potential future prognosis for ISFT.

#### 3.4.3. Follow-up

Follow-up and monitoring of SFT are inevitable recommendations (51). According to the research, long-term persistence in follow-up is rare among patients. Approximately 80% of the patients received follow-up. It is believed that the stable condition obtained by the patient is positively connected to uninterruptible follow-up. In the future, reasonably close monitoring and long-term follow-up would be provided to patients so that their prognosis can be improved.

## 4. Conclusion

ISFT is a very rare intracranial soft tissue tumor that has a high rate of recurrence, metastasis, and death. Currently, pathology and immunohistochemistry are applied in diagnosis. When it comes to treatment, surgical excision is the primary treatment mode, but radiotherapy, targeted therapy, and immunotherapy may be more effective when combined. As SFT survival times increase, it is imperative to maintain close long-term follow-up. Furthermore, tumor “malignancy” seems to be a possible transformation for some patients. Therefore, further investigation is needed to deduce whether a more aggressive approach to tumor management is needed.

## Data availability statement

The original contributions presented in the study are included in the article/Supplementary material, further inquiries can be directed to the corresponding authors.

## Ethics statement

Written informed consent was obtained from the individual(s) for the publication of any potentially identifiable images or data included in this article.

## Author contributions

YG, YX, and RX undertook conceptualization, organizing figures, writing and revising, and original manuscript. DX and YS contributed to composing the patient's medical history. ZZ and JL revised the manuscript and collected the data. TH and ZP contributed to administrative, technical and material support, writing, review, and editing. All authors contributed to the article and approved the submitted version.

## References

[1] Huang SC Huang HY. Solitary fibrous tumor: an evolving and unifying entity with unsettled issues. Histol Histopathol. (2019) 34:313–34. 10.14670/HH-18-064 30431144

[2] Aljohani HT Chaussemy D Proust F Chibbaro S. Intracranial solitary fibrous tumor/hemangiopericytoma: report of two cases and literature review. Int J Health Sci (Qassim). (2017) 11:69–70. 28936155 PMC5604277

[3] Kazazian K Demicco EG de Perrot M Strauss D Swallow CJ. Toward better understanding and management of solitary fibrous tumor. Surg Oncol Clin N Am. (2022) 31:459–83. 10.1016/j.soc.2022.03.009 35715145

[4] Liu Y Wang Q Zhang T Yang L Liang WJ MR. imaging of intracranial solitary fibrous tumor: a retrospective study of 7 cases. Afr Health Sci. (2018) 18:799–806. 10.4314/ahs.v18i3.39 30603014 PMC6306993

[5] Zhao LN Yuan JP Ren JC He HH. Zhonghua Bing Li Xue Za Zhi. (2022) 51:366–8. 10.3760/cma.j.cn112151-20210831-00649 35359054

[6] Giordan E Marton E Wennberg AM Guerriero A Canova G. A review of solitary fibrous tumor/hemangiopericytoma tumor and a comparison of risk factors for recurrence, metastases, and death among patients with spinal and intracranial tumors. Neurosurg Rev. (2021) 44:1299–312. 10.1007/s10143-020-01335-x 32556679

[7] Ronchi A Cozzolino I Marino FZ Accardo M Montella M Panarese I. Extrapleural solitary fibrous tumor: a distinct entity from pleural solitary fibrous tumor. An update on clinical, molecular and diagnostic features. Ann Diagn Pathol. (2018) 34:142–50. 10.1016/j.anndiagpath.2018.01.004 29660566

[8] Bauer JL Miklos AZ Thompson LD. Parotid gland solitary fibrous tumor: a case report and clinicopathologic review of 22 cases from the literature. Head Neck Pathol. (2012) 6:21–31. 10.1007/s12105-011-0305-8 22002440 PMC3311954

[9] Stout AP Murray MR. Hemangiopericytoma: a vascular tumor featuring Zimmermann's pericytes. Ann Surg. (1942) 116:26–33. 10.1097/00000658-194207000-00004 17858068 PMC1543753

[10] Louis DN Ohgaki H Wiestler OD. The 2007 WHO classification of tumours of the central nervous system. Acta Neuropathol. (2007) 114:97–109. 10.1007/s00401-007-0243-4 17618441 PMC1929165

[11] Coindre JM. Nouvelle classification de l'OMS des tumeurs des tissus mous et des os [New WHO classification of tumours of soft tissue and bone]. Ann Pathol. (2012) 32:S115–6. 10.1016/j.annpat.2012.07.006 23127926

[12] Schweizer L Koelsche C Sahm F Piro RM Capper D Reuss DE. Meningeal hemangiopericytoma and solitary fibrous tumors carry the NAB2-STAT6 fusion and can be diagnosed by nuclear expression of STAT6 protein. Acta Neuropathol. (2013) 125:651–8. 10.1007/s00401-013-1117-6 23575898

[13] Louis DN Perry A Reifenberger G von Deimling A Figarella-Branger D Cavenee WK. The 2016 World Health Organization Classification of Tumors of the Central Nervous System: a summary. Acta Neuropathol. (2016) 131:803–20. 10.1007/s00401-016-1545-1 27157931

[14] Louis DN Perry A Wesseling P Brat DJ Cree IA Figarella-Branger D. The 2021 WHO classification of tumors of the central nervous system: a summary. Neuro Oncol. (2021) 23:1231–51. 10.1093/neuonc/noab106 34185076 PMC8328013

[15] Yin H Ye D Zhu Y Geng C. Solitary Fibrous Tumor Of The Great Omentum: A Case Report And Literature Review. Curr Med Imaging. (2022) 18:417–20. 10.2174/1573405617666211108111624 34749624

[16] Yoshida A Tsuta K Ohno M Yoshida M Narita Y Kawai A. STAT6 immunohistochemistry is helpful in the diagnosis of solitary fibrous tumors. Am J Surg Pathol. (2014) 38:552–9. 10.1097/PAS.0000000000000137 24625420

[17] Gubian A Ganau M Cebula H Todeschi J Scibilia A Noel G. Intracranial solitary fibrous tumors: a heterogeneous entity with an uncertain clinical behavior. World Neurosurg. (2019) 126:e48–56. 10.1016/j.wneu.2019.01.142 30716501

[18] Zhao XY Zeng M Yang QY Jing CP Zhang Y. Scrotum solitary fibrous tumor: a case report and review of literature. Medicine. (2017) 96:e8854. 10.1097/MD.0000000000008854 29310366 PMC5728767

[19] Wang ZY Qiu K Ma YH Wang XT Bao JJ Zhang ZF. Intracranial solitary fibrous tumors: a report of two cases and a review of the literature. Oncol Lett. (2016) 11:1057–60. 10.3892/ol.2015.3985 26893690 PMC4734070

[20] Hong J-H Noh M-G Akanda MR Kim YJ Kim SH Jung T-Y. Solitary fibrous tumor/hemangiopericytoma metastasizes extracranially, associated with altered expression of WNT5A and MMP9. Cancers. (2021) 13:1142. 10.3390/cancers13051142 33799999 PMC7962064

[21] Ratneswaren T Hogg FRA Gallagher MJ Ashkan K. Surveillance for metastatic hemangiopericytoma-solitary fibrous tumors-systematic literature review on incidence, predictors and diagnosis of extra-cranial disease. J Neurooncol. (2018) 138:447–67. 10.1007/s11060-018-2836-2 29551003

[22] Melone AG D'Elia A Santoro F Salvati M Delfini R Cantore G. Intracranial hemangiopericytoma–our experience in 30 years: a series of 43 cases and review of the literature. World Neurosurg. (2014) 81:556–62. 10.1016/j.wneu.2013.11.009 24239740

[23] Schiariti M Goetz P El-Maghraby H Tailor J Kitchen N. Hemangiopericytoma: long-term outcome revisited. Clinical article J Neurosurg. (2011) 114:747–55. 10.3171/2010.6.JNS091660 20672899

[24] Chen LF Yang Y Yu XG Gui QP Xu BN Zhou DB. Multimodal treatment and management strategies for intracranial hemangiopericytoma. J Clin Neurosci. (2015) 22:718–25. 10.1016/j.jocn.2014.11.011 25744076

[25] Damodaran O Robbins P Knuckey N Bynevelt M Wong G Lee G. Primary intracranial haemangiopericytoma: comparison of survival outcomes and metastatic potential in WHO grade II and III variants. J Clin Neurosci. (2014) 21:1310–4. 10.1016/j.jocn.2013.11.026 24726230

[26] Dermawan JK Rubin BP Kilpatrick SE. CD34-negative solitary fibrous tumor: a clinicopathologic study of 25 cases and comparison with their CD34-positive counterparts. Am J Surg Pathol. (2021) 45:1616–25. 10.1097/PAS.0000000000001717 34152108

[27] Oliveira E Guerreiro F Lavrador JP. Is stereotactic radiosurgery a treatment option for intracranial solitary fibrous tumors? J Neurosurg Sci. (2017) 61:442–4. 10.23736/S0390-5616.16.03820-0 28555487

[28] Shirakura T Yamada Y Nakata S Asayama B Seo Y Tanikawa S. Analysis of clinicopathological features and NAB2-STAT6 fusion variants of meningeal solitary fibrous tumor with ectopic salivary gland components in the cerebellopontine angle. Virchows Arch. (2022) 481:913–23. 10.1007/s00428-022-03403-7 36056239

[29] Lottin M Escande A Bauchet L Albert-Thananayagam M Barthoulot M Peyre M. Intracranial solitary fibrous tumour management: a french multicentre retrospective study. Cancers. (2023) 15:704. 10.3390/cancers15030704 36765662 PMC9913492

[30] Kim BS Kim Y Kong DS Nam D-H Lee JI Suh YL. Clinical outcomes of intracranial solitary fibrous tumor and hemangiopericytoma: analysis according to the 2016 WHO classification of central nervous system tumors. J Neurosurg. (2018) 129:1384–96. 10.3171/2017.7.JNS171226 29372881

[31] Bisceglia M Galliani C Giannatempo G Lauriola W Bianco M D'angelo V. Solitary fibrous tumor of the central nervous system: a 15-year literature survey of 220 cases (August 1996-July 2011). Adv Anat Pathol. (2011) 18:356–92. 10.1097/PAP.0b013e318229c004 21841406

[32] Gou Q Xie Y Ai P. Intracranial solitary fibrous tumor/hemangiopericytoma: role and choice of postoperative radiotherapy techniques. Front Oncol. (2022) 12:994335. 10.3389/fonc.2022.994335 36249022 PMC9554559

[33] Allen AJ Labella DA Richardson KM Sheehan JP Kersh CR. Recurrent solitary fibrous tumor (Intracranial Hemangiopericytoma) treated with a novel combined-modality radiosurgery technique: a case report and review of the literature. Front Oncol. (2022) 12:907324. 10.3389/fonc.2022.907324 35720016 PMC9204631

[34] Kano H Niranjan A Kondziolka D Flickinger JC Lunsford LD. Adjuvant stereotactic radiosurgery after resection of intracranial hemangiopericytomas. Int J Radiat Oncol Biol Phys. (2008) 72:1333–9. 10.1016/j.ijrobp.2008.03.024 18723295

[35] Tsugawa T Mori Y Kobayashi T Hashizume C Shibamoto Y Wakabayashi T. Gamma knife stereotactic radiosurgery for intracranial hemangiopericytoma. J Radiosurg SBRT. (2014) 3:29–35. 29296382 PMC5725327

[36] Cohen-Inbar O Lee C-C Mousavi SH Kano H Mathieu D Meola A. Stereotactic radiosurgery for intracranial hemangiopericytomas: a multicenter study. J Neurosurg. (2017) 126:744–54. 10.3171/2016.1.JNS152860 27104850

[37] Olson C Yen CP Schlesinger D Sheehan J. Radiosurgery for intracranial hemangiopericytomas: outcomes after initial and repeat Gamma Knife surgery. J Neurosurg. (2010) 112:133–9. 10.3171/2009.3.JNS0923 19392594

[38] Park MS Ravi V Conley A Patel SR Trent JC Lev DC. The role of chemotherapy in advanced solitary fibrous tumors: a retrospective analysis. Clin Sarcoma Res. (2013) 3:7. 10.1186/2045-3329-3-7 23663788 PMC3660184

[39] Constantinidou A Jones RL Olmos D Thway K Fisher C Al-Muderis O. Conventional anthracycline-based chemotherapy has limited efficacy in solitary fibrous tumour. Acta Oncol. (2012) 51:550–4. 10.3109/0284186X.2011.626450 22023088

[40] Park MS Patel SR Ludwig JA Trent JC Conrad CA Lazar AJ. Activity of temozolomide and bevacizumab in the treatment of locally advanced, recurrent, and metastatic hemangiopericytoma and malignant solitary fibrous tumor. Cancer. (2011) 117:4939–47. 10.1002/cncr.26098 21480200 PMC3135685

[41] Martin-Broto J Stacchiotti S Lopez-Pousa A Redondo A Bernabeu D de Alava E. Pazopanib for treatment of advanced malignant and dedifferentiated solitary fibrous tumour: a multicentre, single-arm, phase 2 trial. Lancet Oncol. (2019) 20:134–44. 10.1016/S1470-2045(18)30676-4 30578023

[42] Stacchiotti S Negri T Libertini M Palassini E Marrari A De Troia B. Sunitinib malate in solitary fibrous tumor (SFT). Ann Oncol. (2012) 23:3171–9. 10.1093/annonc/mds143 22711763

[43] Shen G Zheng F Ren D. Anlotinib: a novel multi-targeting tyrosine kinase inhibitor in clinical development. J Hematol Oncol. (2018) 11:120. 10.1186/s13045-018-0664-7 30231931 PMC6146601

[44] Li S. Anlotinib: a novel targeted drug for bone and soft tissue sarcoma. Front Oncol. (2021) 11:664853. 10.3389/fonc.2021.664853 34094958 PMC8173120

[45] Yuan M Zhu Z Mao W. Anlotinib Combined With Anti-PD-1 antibodies therapy in patients with advanced refractory solid tumors: a single-center, observational, prospective study. Front Oncol. (2021) 11:683502. 10.3389/fonc.2021.683502 34692475 PMC8529018

[46] Li S Zhang B Zhang P Xue C Deng J Liu X. Postoperative progression of intracranial grade II-III solitary fibrous tumor/hemangiopericytoma: predictive value of preoperative magnetic resonance imaging semantic features. Acta Radiol. (2021) 3:2841851211066757. 10.1177/02841851211066757 34923852

[47] Apra C Mokhtari K Cornu P Peyre M Kalamarides M. Intracranial solitary fibrous tumors/hemangiopericytomas: first report of malignant progression. J Neurosurg. (2018) 128:1719–24. 10.3171/2017.1.JNS162593 28644098

[48] Kamamoto D Ohara K Kitamura Y Yoshida K Kawakami Y Sasaki H. Association between programmed cell death ligand-1 expression and extracranial metastasis in intracranial solitary fibrous tumor/hemangiopericytoma. J Neurooncol. (2018) 139:251–9. 10.1007/s11060-018-2876-7 29675794

[49] Lavacchi D Antonuzzo L Briganti V Berti V Abenavoli EM Linguanti F. Metastatic intracranial solitary fibrous tumors/hemangiopericytomas: description of two cases with radically different behaviors and review of the literature. Anticancer Drugs. (2020) 31:646–51. 10.1097/CAD.0000000000000900 31972591

[50] Liu J Wu S Zhao K Wang J Shu K Lei T. Clinical features, management, and prognostic factors of intracranial solitary fibrous tumor. Front Oncol. (2022) 12:915273. 10.3389/fonc.2022.915273 35712477 PMC9197442

[51] Swaminathan S Ruzevick J Venur V Halasz LM Rockhill J Gonzalez-Cuyar L. Intracranial solitary fibrous tumor/hemangiopericytoma treated with microsurgical resection: retrospective cohort analysis of a single-center experience. Ther Clin Risk Manag. (2022) 18:901–12. 10.2147/TCRM.S375064 36092453 PMC9462835

