# Supplementary figures and images for: A rare case of intracranial solitary fibrous tumor that is still alive after multiple surgical resections: a case report and review of the literature
Source: Front Neurol. 2023 Jul 10;14:1201964. doi: 10.3389/fneur.2023.1201964 (PMC10363678; doi:10.3389/fneur.2023.1201964)

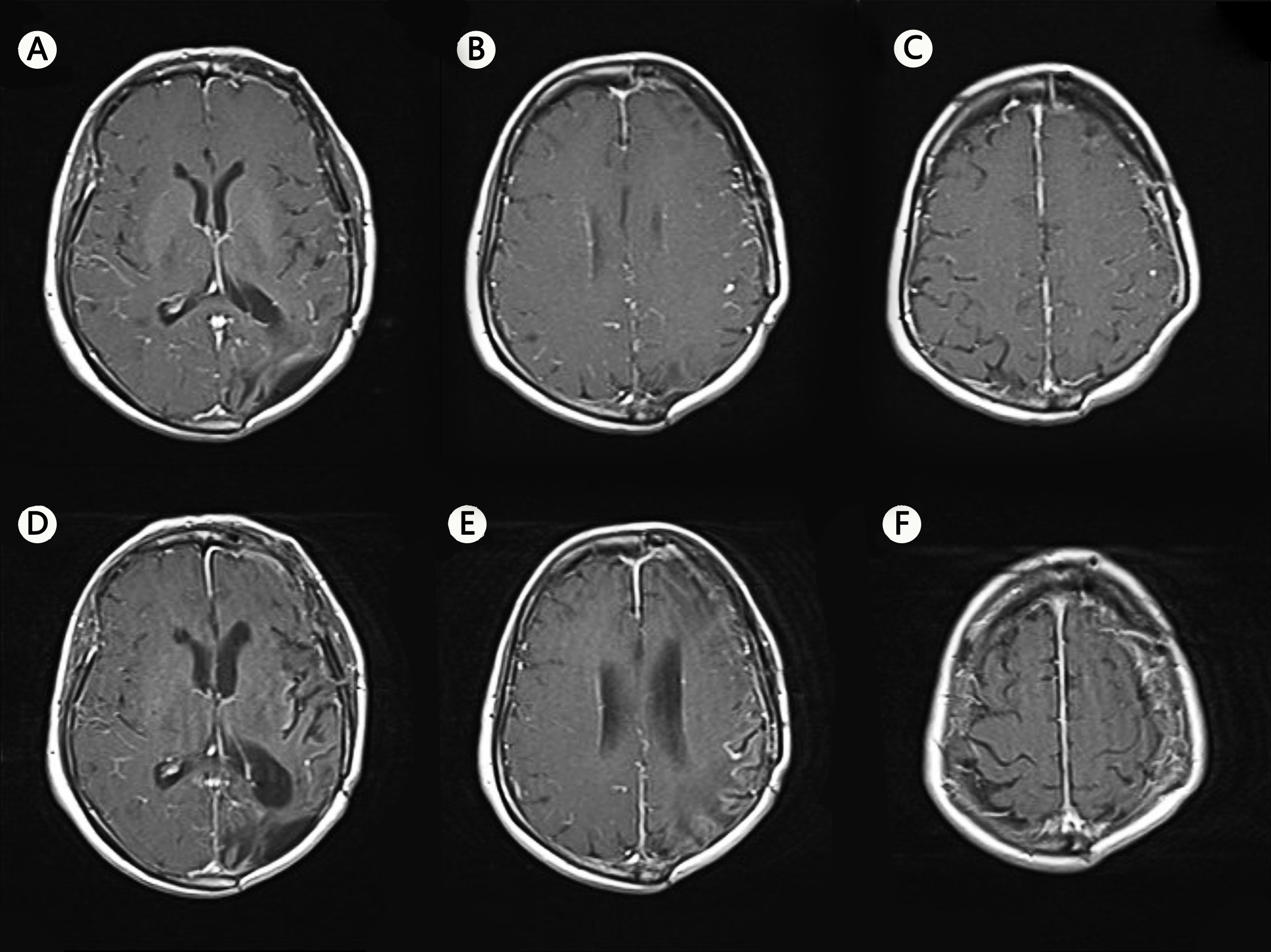

Supplement: Supplementary file 1 [file Image_1.JPEG]
